# Supplementary material for: Immune-Related Genes in the Honey Bee Mite Varroa destructor (Acarina, Parasitidae)
Source: Insects. 2025 Mar 28;16(4):356. doi: 10.3390/insects16040356 (PMC12027997; doi:10.3390/insects16040356)
Supplement: Supplementary file 1 [file insects-16-00356-s001.zip › Table S1.pdf]

Table S1. Immune genes involved in recognition, signaling and response in *Ixodes scapularis*

Gene Name	Role	Gene Symbol	Ixodes scapularis	Drosophila melanogaster	E-Value	identity	Coverage
**Immune genes involved in recognition**							
peptidoglycan recognition protein	bacterial recognition	PGRP-LC	EEC16399.1	AAF50302.3	2e-46	47.40 %	30%
peptidoglycan recognition protein	activation of PPO cascade and autophagy	PGRP-LE	XP_002413091.3	NP_573078.1	3e-39	42.86 %	46%
peptidoglycan recognition protein	bacterial recognition	PGRP-SA	XP_002413091.3	AAF48056.1	5e-40	36.46 %	92%
peptidoglycan recognition protein	bacterial recognition	PGRP-SD	EEC16399.1	CAD89193.1	1e-40	36.02 %	98%
peptidoglycan recognition protein	bacterial recognition	PGRP-LB	XP_002413091.3	NP_650079.1	2e-41	41.36 %	74%
peptidoglycan recognition protein	bacterial recognition	PGRP-SC1a	EEC16399.1	CAD89161.1	4e-52	41.76 %	97%
peptidoglycan recognition protein	bacterial recognition	PGRP-SC2	XP_002413091.3	CAD89187.1	3e-51	42.70 %	89%
peptidoglycan recognition protein	pgn degradation and antibacterial activity	PGRP-SB1	XP_002413091.3	CAD89136.1	8e-46	42.61 %	92%
peptidoglycan recognition protein	blocking of imd pathway	PGRP-LF	EEC16399.1	NP_648299.3	1e-38	34.76 %	79%
peptidoglycan recognition protein	*activation of imd pathway*	*PGRP-LA*	*Not found*	*AAF50304.2*	*Not found*	*Not found*	*Not found*
Gram-negative binding protein 1	*bacterial and fungal pattern recognition*	*GNBP1*	*Not found*	*Q9NHB0.2*	*Not found*	*Not found*	*Not found*
Gram-negative binding protein 2	*bacterial and fungal pattern recognition*	*GNBP2*	*Not found*	*ACU30172.1*	*Not found*	*Not found*	*Not found*
Gram-negative binding protein 3	*bacterial and fungal pattern recognition*	*GNBP3*	*Not found*	*CAJ18910.1*	*Not found*	*Not found*	*Not found*
c-type lectin 1	*bacterial recognition, induction of PPO cascade*	*DL1*	*Not found*	*AAF53793.1*	*Not found*	*Not found*	*Not found*

c-type lectin 2	*bacterial recognition, induction of PPO cascade*	*DL2*	*Not found*	*NP_001014489.1*	*Not found*	*Not found*	*Not found*
c-type lectin 3 or solute carrier	*bacterial recognition, induction of PPO cascade*	*DL3*	*Not found*	*NP_001014490.1*	*Not found*	*Not found*	*Not found*
galectin 4	several roles have been hypothesized	galectin	XP_029832035.2	ADZ99399.1	3e-29	28.23 %	82%
CD109 antigen-like	mark pathogens for phagocytosis	Tep1	XP_040071930.1	CAB87807.1	0.0	32.51 %	99%
CD109 antigen-like	mark pathogens	Tep2	XP_040071930.1	CAB87808.1	0.0	36.74 %	98%
CD109 antigen-like	mark pathogens	Tep3	XP_040071930.1	AAL39195.1	0.0	34.87 %	96%
CD109 antigen-like	mark pathogens	Tep4	XP_040071930.1	NP_523603.2	0.0	31.85 %	99%
scavenger receptor class B member 1-like	bacterial and fungal recognition	pes	XP_029842404.2	AHN54246.1	3e-74	31.57 %	82%
lysosome membrane protein 2-like	bacterial and fungal recognition	crq	XP_029842404.2	AAF51494.1	1e-82	31.27 %	98%
protein draper-like	bacterial and fungal recognition	drpr	XP_042142012.1	NP_477450.1	7e-92	38.89 %	88%
scavenger receptor class c, type i	bind to lipoproteins and bacteria	sr-CI	XP_040358695.2	AAW79470.1	5e-23	29.74 %	51%
scavenger receptor class c, type ii	bind to lipoproteins and bacteria	sr-CII	XP_040358695.2	AAF58551.1	1e-19	28.57 %	48%
scavenger receptor class c, type iii	bind to lipoproteins and bacteria	sr-CIII	XP_040358695.2	AAF37564.1	1e-15	25.49 %	91%
scavenger receptor class c, type iv	bind to lipoproteins and bacteria	sr-CIV	XP_040358695.2	AAF51092.1	4e-23	29.74 %	64%
eater	receptor in phagocytosis	eater	XP_042147136.1	AAF56664.5	6e-20	36.73 %	73%

	and microbial binding						
protein draper-like/Nimrod	receptor in phagocytosis and microbial binding	Drp/Nim	XP_040079451.2	AAF53364.2	2e-18	29.71%	65%
**Immune genes involved in Signaling**							
spätzle 1B	Toll pathway	spz1-1	XP_040074202.1	NP_733188.1	2e-14	34.21%	49%
spätzle 1Bii	Toll pathway	spz1-2	XP_040074202.1	NP_001138116.1	7e-14	34.21%	41%
spätzle 2, neurotrophin 1	Toll pathway	Spz2	XP_040075619.3	NP_001261417.1	2e-19	28.50%	16%
spätzle 3	Toll pathway	Spz3	XP_040068871.1	NP_609160.2	4e-74	46.96	40%
spätzle 4	Toll pathway	Spz4	XP_040354538.2	NP_609504.2	5e-55	78.35%	54%
spätzle 5	Toll pathway	Spz5	XP_040074202.1	NP_647753.1	3e-16	32.84%	33%
spätzle 6	Toll pathway	Spz6	EEC04158.1	NP_611961.1	5e-59	53.85%	85%
protein Toll	Toll pathway	Toll-1	XP_029836730.1	NP_524518.1	4e-123	30.38%	86%
protein Toll	Toll pathway	Toll-1	EEC04824.1	NP_524518.1	2e-122	30.28%	86%
18 wheeler, Toll-2	Toll pathway	18w	EEC15829.1	NP_476814.1	0.0	40.60%	83%
Toll-6	Toll pathway	Toll-6	XP_029821922.2	NP_001246766.1	0.0	42.74%	77%
Toll-6	Toll pathway	Toll-6	XP_040077612.1	NP_001246766.1	0.0	41.41%	77%
Toll-7	Toll pathway	Toll-7	EEC15829.1	NP_523797.1	0.0	41.98%	80%
Tollo, Toll-8	Toll pathway	Tollo	EEC15829.1	NP_524757.1	0.0	43.11%	89%
tube, interleukin-1 receptor-associated kinase 4	*Toll pathway*	*tub*	*Not found*	*NP_001189164.1*	*Not found*	*Not found*	*Not found*
myeloid differentiation primary response gene	Toll pathway	Myd88pll	EEC12280.1	AAF58953.1	5e-19	25.56%	48%
pelle	Toll pathway	pll	EEC01388.1	AAF56686.1	4e-78	49.83%	58%
cactus	Toll pathway	cact	XP_029826582.3	AAN10936.1	6e-29	34.73%	45%
cactin	Toll pathway	cactin	EEC06953.1	NP_523422.4	0.0	50.61	85%

						%	
pellino	Toll pathway	Pli	XP_029830098.1	NP_524466.1	0.0	69.48 %	94%
TNF-receptor-associated factor 1	Toll pathway	Traf1, Traf4	XP_040061820.1	AAD34346.1	1e-124	53.68 %	66%
TNF-receptor-associated factor 2	Toll pathway	Traf2, Traf6	XP_002412592.3	AAF46338.1	1e-15	20.43 %	80%
TNF-receptor-associated factor 3	Toll pathway	Traf3, Traf-like	XP_040061820.1	NP_727976.1	3e-16	26.34 %	44%
dorsal	Toll pathway	dl	XP_029825506.2	AAF53611.1	3e-116	57.05 %	44%
domeless 1, interleukine JAK/STAT receptor	Jak/stat pathway	dome	XP_029844459.2	CAD12503.1	2e-21	22.24 %	38%
Domeless2	Jak/stat pathway	dome2	XP_029844432.3	XP_029341036.1	1e-44	25.56 %	33%
hopscotch, Janus kinas	Jak/stat pathway	hops, jak	EEC01610.1	NP_511119.2	6e-55	44.70 %	21%
signal-transducer and activator of transcription, marelle	Jak/stat pathway	Stat92E	XP_040069485.1	AAX33462.1	6e-137	36.94 %	94%
unpaired 1	*Jak/stat pathway*	*upd1*	*Not found*	*NP_525095.2*	*Not found*	*Not found*	*Not found*
unpaired 2	*Jak/stat pathway*	*Upd2*	*Not found*	*NP_001356882.1*	*Not found*	*Not found*	*Not found*
unpaired 3	*Jak/stat pathway*	*Upd3*	*Not found*	*NP_001097014.1*	*Not found*	*Not found*	*Not found*
immune deficiency	*Imd pathway*	*imd*	*Not found*	*NP_573394.1*	*Not found*	*Not found*	*Not found*
dFadd	*Imd pathway*	*dFadd*	*Not found*	*NP_651006.1*	*Not found*	*Not found*	*Not found*
death related ced-3, caspase-1	Imd pathway	Dredd	XP_040068356.1	NP_477249.3	2e-18	29.68 %	41%
Relish	Imd pathway	Rel	XP_040356997.1	NP_477094.1	8e-52	37.33 %	34%
TAK1-associated binding protein 2	Imd pathway	Tab2	EEC12297.1	NP_611408.2	3e-04	53.33 %	3%
TGF-β activated Kinase 1	Imd pathway	Tak1	XP_029850224.1	AAF50895.1	1e-100	33.70 %	90%
kenny	Imd pathway	key	XP_040356845.1	NP_523856.2	6e-04	28.24 %	33%

death-associated inhibitor of apoptosis 2	Imd pathway	Diap2	XP_029832541.2	NP_477127.1	4e-84	32.96 %	99%
immune response deficiency 5, IK-β, IKKB, I-kappaB kinase beta	Imd pathway	ird5	XP_029847730.2	NP_524751.3	2e-46	32.82 %	45%
hemipterous	Jnk pathway	hep	XP_002408117.3	NP_727661.1	8e-119	57.51 %	26%
basket	Jnk pathway	bsk	XP_002408829.1	P92208.1	0.0	85.56 %	96%
Jun-related antigen	Jnk pathway	Jra	XP_029831021.2	AAF58845.1	9e-46	41.28 %	78%
kayak	Jnk pathway	kay	EEC13979.1	NP_001027579.1	6e-21	56.67 %	23%
Eiger	*Jnk pathway*	*egr*	*Not found*	*AAF58848.2*	*Not found*	*Not found*	*Not found*
spätzle 1B	Toll pathway	spz1-1	XP_040074202.1	NP_733188.1	2e-14	34.21 %	49%
spätzle 1Bii	Toll pathway	spz1-2	XP_040074202.1	NP_001138116.1	7e-14	34.21 %	41%
spätzle 2, neurotrophin 1	Toll pathway	Spz2	XP_040075619.3	NP_001261417.1	2e-19	28.50 %	16%
spätzle 3	Toll pathway	Spz3	XP_040068871.1	NP_609160.2	4e-74	46.96	40%
spätzle 4	Toll pathway	Spz4	XP_040354538.2	NP_609504.2	5e-55	78.35 %	54%
spätzle 5	Toll pathway	Spz5	XP_040074202.1	NP_647753.1	3e-16	32.84 %	33%
spätzle 6	Toll pathway	Spz6	EEC04158.1	NP_611961.1	5e-59	53.85 %	85%
protein Toll	Toll pathway	Toll-1	XP_029836730.1	NP_524518.1	4e-123	30.38 %	86%
protein Toll	Toll pathway	Toll-1	EEC04824.1	NP_524518.1	2e-122	30.28 %	86%
18 wheeler, Toll-2	Toll pathway	18w	EEC15829.1	NP_476814.1	0.0	40.60 %	83%
Toll-6	Toll pathway	Toll-6	XP_029821922.2	NP_001246766.1	0.0	42.74 %	77%
Toll-6	Toll pathway	Toll-6	XP_040077612.1	NP_001246766.1	0.0	41.41 %	77%
Toll-7	Toll pathway	Toll-7	EEC15829.1	NP_523797.1	0.0	41.98 %	80%
Tollo, Toll-8	Toll pathway	Tollo	EEC15829.1	NP_524757.1	0.0	43.11 %	89%
tube, interleukin-1 receptor-	*Toll pathway*	*tub*	*Not found*	*NP_001189164.1*	*Not found*	*Not found*	*Not found*

associated kinase 4							
myeloid differentiation primary response gene	Toll pathway	Myd88pll	EEC12280.1	AAF58953.1	5e-19	25.56 %	48%
pelle	Toll pathway	pll	EEC01388.1	AAF56686.1	4e-78	49.83 %	58%
cactus	Toll pathway	cact	XP_029826582.3	AAN10936.1	6e-29	34.73 %	45%
cactin	Toll pathway	cactin	EEC06953.1	NP_523422.4	0.0	50.61 %	85%
pellino	Toll pathway	Pli	XP_029830098.1	NP_524466.1	0.0	69.48 %	94%
TNF-receptor-associated factor 1	Toll pathway	Traf1, Traf4	XP_040061820.1	AAD34346.1	1e-124	53.68 %	66%
TNF-receptor-associated factor 2	Toll pathway	Traf2, Traf6	XP_002412592.3	AAF46338.1	1e-15	20.43 %	80%
TNF-receptor-associated factor 3	Toll pathway	Traf3, Traf-like	XP_040061820.1	NP_727976.1	3e-16	26.34 %	44%
dorsal	Toll pathway	dl	XP_029825506.2	AAF53611.1	3e-116	57.05 %	44%
domeless 1, interleukine JAK/STAT receptor	Jak/stat pathway	dome	XP_029844459.2	CAD12503.1	2e-21	22.24 %	38%
Domeless2	Jak/stat pathway	dome2	XP_029844432.3	XP_029341036.1	1e-44	25.56 %	33%
hopscotch, Janus kinas	Jak/stat pathway	hops, jak	EEC01610.1	NP_511119.2	6e-55	44.70 %	21%
signal-transducer and activator of transcription, marelle	Jak/stat pathway	Stat92E	XP_040069485.1	AAX33462.1	6e-137	36.94 %	94%
unpaired 1	*Jak/stat pathway*	*upd1*	*Not found*	*NP_525095.2*	*Not found*	*Not found*	*Not found*
unpaired 2	*Jak/stat pathway*	*Upd2*	*Not found*	*NP_001356882.1*	*Not found*	*Not found*	*Not found*
unpaired 3	*Jak/stat pathway*	*Upd3*	*Not found*	*NP_001097014.1*	*Not found*	*Not found*	*Not found*
immune deficiency	*Imd pathway*	*imd*	*Not found*	*NP_573394.1*	*Not found*	*Not found*	*Not found*
dFadd	*Imd pathway*	*dFadd*	*Not found*	*NP_651006.1*	*Not found*	*Not found*	*Not found*
death related ced-3, caspase-1	Imd pathway	Dredd	XP_040068356.1	NP_477249.3	2e-18	29.68 %	41%

Relish	Imd pathway	Rel	XP_040356997.1	NP_477094.1	8e-52	37.33 %	34%
TAK1-associated binding protein 2	Imd pathway	Tab2	EEC12297.1	NP_611408.2	3e-04	53.33 %	3%
TGF-β activated Kinase 1	Imd pathway	Tak1	XP_029850224.1	AAF50895.1	1e-100	33.70 %	90%
kenny	Imd pathway	key	XP_040356845.1	NP_523856.2	6e-04	28.24 %	33%
death-associated inhibitor of apoptosis 2	Imd pathway	Diap2	XP_029832541.2	NP_477127.1	4e-84	32.96 %	99%
immune response deficiency 5, IK-β, IKKB, I-kappaB kinase beta	Imd pathway	ird5	XP_029847730.2	NP_524751.3	2e-46	32.82 %	45%
hemipterous	Jnk pathway	hep	XP_002408117.3	NP_727661.1	8e-119	57.51 %	26%
basket	Jnk pathway	bsk	XP_002408829.1	P92208.1	0.0	85.56 %	96%
Jun-related antigen	Jnk pathway	Jra	XP_029831021.2	AAF58845.1	9e-46	41.28 %	78%
kayak	Jnk pathway	kay	EEC13979.1	NP_001027579.1	6e-21	56.67 %	23%
Eiger	*Jnk pathway*	*egr*	*Not found*	*AAF58848.2*	*Not found*	*Not found*	*Not found*
spätzle 1B	Toll pathway	spz1-1	XP_040074202.1	NP_733188.1	2e-14	34.21 %	49%
spätzle 1Bii	Toll pathway	spz1-2	XP_040074202.1	NP_001138116.1	7e-14	34.21 %	41%
spätzle 2, neurotrophin 1	Toll pathway	Spz2	XP_040075619.3	NP_001261417.1	2e-19	28.50 %	16%
spätzle 3	Toll pathway	Spz3	XP_040068871.1	NP_609160.2	4e-74	46.96	40%
spätzle 4	Toll pathway	Spz4	XP_040354538.2	NP_609504.2	5e-55	78.35 %	54%
spätzle 5	Toll pathway	Spz5	XP_040074202.1	NP_647753.1	3e-16	32.84 %	33%
spätzle 6	Toll pathway	Spz6	EEC04158.1	NP_611961.1	5e-59	53.85 %	85%
protein Toll	Toll pathway	Toll-1	XP_029836730.1	NP_524518.1	4e-123	30.38 %	86%
protein Toll	Toll pathway	Toll-1	EEC04824.1	NP_524518.1	2e-122	30.28 %	86%
18 wheeler, Toll-2	Toll pathway	18w	EEC15829.1	NP_476814.1	0.0	40.60 %	83%

Toll-6	Toll pathway	Toll-6	XP_029821922.2	NP_001246766.1	0.0	42.74 %	77%
Toll-6	Toll pathway	Toll-6	XP_040077612.1	NP_001246766.1	0.0	41.41 %	77%
Toll-7	Toll pathway	Toll-7	EEC15829.1	NP_523797.1	0.0	41.98 %	80%
Tollo, Toll-8	Toll pathway	Tollo	EEC15829.1	NP_524757.1	0.0	43.11 %	89%
tube, interleukin-1 receptor-associated kinase 4	*Toll pathway*	*tub*	*Not found*	*NP_001189164.1*	*Not found*	*Not found*	*Not found*
myeloid differentiation primary response gene	Toll pathway	Myd88pll	EEC12280.1	AAF58953.1	5e-19	25.56 %	48%
pelle	Toll pathway	pll	EEC01388.1	AAF56686.1	4e-78	49.83 %	58%
cactus	Toll pathway	cact	XP_029826582.3	AAN10936.1	6e-29	34.73 %	45%
cactin	Toll pathway	cactin	EEC06953.1	NP_523422.4	0.0	50.61 %	85%
pellino	Toll pathway	Pli	XP_029830098.1	NP_524466.1	0.0	69.48 %	94%
TNF-receptor-associated factor 1	Toll pathway	Traf1, Traf4	XP_040061820.1	AAD34346.1	1e-124	53.68 %	66%
TNF-receptor-associated factor 2	Toll pathway	Traf2, Traf6	XP_002412592.3	AAF46338.1	1e-15	20.43 %	80%
TNF-receptor-associated factor 3	Toll pathway	Traf3, Traf-like	XP_040061820.1	NP_727976.1	3e-16	26.34 %	44%
dorsal	Toll pathway	dl	XP_029825506.2	AAF53611.1	3e-116	57.05 %	44%
domeless 1, interleukine JAK/STAT receptor	Jak/stat pathway	dome	XP_029844459.2	CAD12503.1	2e-21	22.24 %	38%
Domeless2	Jak/stat pathway	dome2	XP_029844432.3	XP_029341036.1	1e-44	25.56 %	33%
hopscotch, Janus kinas	Jak/stat pathway	hops, jak	EEC01610.1	NP_511119.2	6e-55	44.70 %	21%
signal-transducer and activator of transcription, marelle	Jak/stat pathway	Stat92E	XP_040069485.1	AAX33462.1	6e-137	36.94 %	94%
unpaired 1	*Jak/stat pathway*	*upd1*	*Not found*	*NP_525095.2*	*Not found*	*Not found*	*Not found*

unpaired 2	*Jak/stat pathway*	*Upd2*	*Not found*	*NP_001356882.1*	*Not found*	*Not found*	*Not found*
unpaired 3	*Jak/stat pathway*	*Upd3*	*Not found*	*NP_001097014.1*	*Not found*	*Not found*	*Not found*
immune deficiency	*Imd pathway*	*imd*	*Not found*	*NP_573394.1*	*Not found*	*Not found*	*Not found*
dFadd	*Imd pathway*	*dFadd*	*Not found*	*NP_651006.1*	*Not found*	*Not found*	*Not found*
death related ced-3, caspase-1	Imd pathway	Dredd	XP_040068356.1	NP_477249.3	2e-18	29.68 %	41%
Relish	Imd pathway	Rel	XP_040356997.1	NP_477094.1	8e-52	37.33 %	34%
TAK1-associated binding protein 2	Imd pathway	Tab2	EEC12297.1	NP_611408.2	3e-04	53.33 %	3%
TGF-β activated Kinase 1	Imd pathway	Tak1	XP_029850224.1	AAF50895.1	1e-100	33.70 %	90%
kenny	Imd pathway	key	XP_040356845.1	NP_523856.2	6e-04	28.24 %	33%
death-associated inhibitor of apoptosis 2	Imd pathway	Diap2	XP_029832541.2	NP_477127.1	4e-84	32.96 %	99%
immune response deficiency 5, IK-β, IKKB, I-kappaB kinase beta	Imd pathway	ird5	XP_029847730.2	NP_524751.3	2e-46	32.82 %	45%
hemipterous	Jnk pathway	hep	XP_002408117.3	NP_727661.1	8e-119	57.51 %	26%
basket	Jnk pathway	bsk	XP_002408829.1	P92208.1	0.0	85.56 %	96%
Jun-related antigen	Jnk pathway	Jra	XP_029831021.2	AAF58845.1	9e-46	41.28 %	78%
kayak	Jnk pathway	kay	EEC13979.1	NP_001027579.1	6e-21	56.67 %	23%
Eiger	*Jnk pathway*	*egr*	*Not found*	*AAF58848.2*	*Not found*	*Not found*	*Not found*
**Immune genes involved in response**							
Attacin	*antimicrobial peptide*	*att*	*Not found*	*NP_523745.1*	*Not found*	*Not found*	*Not found*
Cecropin	*antimicrobial peptide*	*Cec*	*Not found*	*C0HKQ7.1*	*Not found*	*Not found*	*Not found*
Defensin	*antimicrobial peptide*	*Def*	*Not found*	*ANY27112.1*	*Not found*	*Not found*	*Not found*

Dosocin	*antimicrobial peptide*	*Dro*	*Not found*	*XP_016946682 .1*	*Not found*	*Not found*	*Not found*
Metchnikowin	*antimicrobial peptide*	*Mtk*	*Not found*	*AAO72489.1*	*Not found*	*Not found*	*Not found*
Andropin	*antimicrobial peptide*		*Not found*	*P21663.1*	*Not found*	*Not found*	*Not found*
Diptericin	*antimicrobial peptide*		*Not found*	*QER92349.1*	*Not found*	*Not found*	*Not found*
drosomycin	*antimicrobial peptide*	*Drs*	*Not found*	*ANY27466.1*	*Not found*	*Not found*	*Not found*
holotricin	*antimicrobial peptide*		*Not found*	*XP_051861657 .1*	*Not found*	*Not found*	*Not found*
bomanin	*antimicrobial peptide*		*Not found*	*A1ZB62.1*	*Not found*	*Not found*	*Not found*
thaumatin-like protein	*antimicrobial*		*Not found*	*XP_001942718 .2*	*Not found*	*Not found*	*Not found*
thaumatin-like protein 1b	*antimicrobial*		*Not found*	*XP_001942572 .1*	*Not found*	*Not found*	*Not found*
thaumatin-like protein 1	*antimicrobial*		*Not found*	*XP_003248856 .4*	*Not found*	*Not found*	*Not found*
uncharacterize d LOC100162111 , thaumatin family	*antimicrobial*		*Not found*	*NP_00115551 6*	*Not found*	*Not found*	*Not found*
TLP-PA-domain protein	*antimicrobial*		*Not found*	*NP_00115630 4.1*	*Not found*	*Not found*	*Not found*
Pathogenesis-related protein 5-like	*antimicrobial*		*Not found*	*NP_00131358 5.1*	*Not found*	*Not found*	*Not found*
lysozyme X, i-type	microbial degradation	LysX	XP_002399 439.3	CAL85493.1	1e-26	39.84 %	90%
lysozyme B, i-type	microbial degradation	LysB	XP_029836 071.4	NP_00126124 5.1	7e-28	42.73 %	77%
lysozyme, i-type	microbial degradation	LysP	EEC00236.1	NP_476828.1	4e-31	45.31 %	90%
Lysozyme E	microbial degradation	LysE	XP_0298360 71.4	CAA80228	6e-28	41.96 %	79%
Lysozyme D	microbial degradation	LysD	XP_0298360 71.4	NP_476823.1	5e-28	41.96 %	79%
Lysozyme E	microbial degradation	LysE	XP_0421477 87.1	NP_476827.2	5e-28	39.39 %	93%
Lysozyme S	microbial degradation	LysS	XP_0298360 71.4	NP_476829.1	2e-26	43.36 %	80%
Lysozyme E	microbial degradation	lysozyme, i-type	XP_0024108 14.3	ACD99447.1	1e-26	38.56 %	83%
Lysozyme	microbial degradation	lysozyme, i-type	EEC13043.1	NP_611164.3	3e-20	33.33 %	91%
Lysozime	microbial degradation	lysozyme, i-type	EEC13043.1	NP_611163.2	1e-22	34.16 %	94%
chitinase-like	fungal	Cht2	XP_0421450	NP_00126128	2e-113	45.39	81%

protein 4, flocculation protein	degradation		90.1	2.1		%	
chitinase-like protein 2, mucin	fungal degradation	Cht4	XP_042145090.1	NP_524962.2	2e-111	46.43 %	74%
chitinase-like protein 5, endochitinase	fungal degradation	Cht5	EEC01930.1	NP_650314.1	1e-164	57.65 %	76%
chitinase-like protein 6, flocculation protein	fungal degradation	Cht6	XP_042150371.1	NP_001245602.1	2e-152	55.47 %	11%
chitinase-like protein 7, chitinase 10	fungal degradation	Cht7	XP_029838643.3	NP_647768.3	0.0	57.74 %	89%
chitinase 3-like	fungal degradation	Cht7	EEC00162.1	NP_647768.3	0.0	55.96 %	89%
Chitinase 6, flocculation protein FLO11	fungal degradation	Cht6	XP_042150371.1	NP_001245599.1	5e-154	55.47 %	31%
idgf	fungal degradation	idgf6	XP_029826461.2	NP_001286499.1	2e-46	29.75 %	98%
prophenoloxidase 1	*prophenoloxidase response*	*PPO1*	*Not found*	*NP_476812.1*	*Not found*	*Not found*	*Not found*
prophenoloxidase 2	*prophenoloxidase response*	*PPO2*	*Not found*	*NP_610443.1*	*Not found*	*Not found*	*Not found*
Phenoloxidase-activating factor 2 (tryptase like)	phenoloxidase activation	PAF2, PPAF2	XP_029825631.3	AAO24923.1	1e-74	45.45 %	64%
Phenoloxidase-activating factor 2	phenoloxidase activation	PAF2, PPAF2	XP_029825533.2	AAO24923.1	2e-73	39.46 %	77%
serine protease-like precursor	phenoloxidase activation	SP	XP_042147178.1	NP_001097766.1	6e-44	38.76 %	56%
hemocytin	cell aggregation	Hmct, hemolectin	XP_042149989.1	NP_001261809.1	0.0	35.26 %	75%
nitric oxide synthase	production of nitric oxide, a toxic gas	Nos	XP_029841849.2	NP_001027243.2	0.0	49.91 %	99%
transglutaminase	clotting	Tg	XP_040062860.3	NP_609174.1	0.0	48.8%	91%
