# Supplementary material for: Immune-Related Genes in the Honey Bee Mite Varroa destructor (Acarina, Parasitidae)
Source: Insects. 2025 Mar 28;16(4):356. doi: 10.3390/insects16040356 (PMC12027997; doi:10.3390/insects16040356)
Supplement: Supplementary file 1 [file insects-16-00356-s001.zip › Table S2.pdf]

Table S2. Immune genes involved in recognition, signaling and response in *Galendromus occidentalis*

Gene Name	Role	Gene Symbol	Galendromus occidentalis	Drosophila melanogaster	E-Value	identity	Coverage
**Immune genes involved in RECOGNITION**							
peptidoglycan recognition protein	bacterial recognition	PGRP-LC	XP_003743951.1	AAF50302.3	7e-29	31.22%	37%
peptidoglycan recognition protein	activation of PPO cascade and autophagy	PGRP-LE	XP_003743951.1	NP_573078.1	3e-21	30.62%	46%
peptidoglycan recognition protein	bacterial recognition	PGRP-SA	XP_003743951.1	AAF48056.1	2e-30	33.33%	82%
peptidoglycan recognition protein	bacterial recognition	PGRP-SD	XP_003743951.1	CAD89193.1	2e-27	31.43%	94%
peptidoglycan recognition protein	bacterial recognition	PGRP-LB	XP_003743951.1	NP_650079.1	7e-30	34.30	80%
peptidoglycan recognition protein	bacterial recognition	PGRP-SC1a	XP_003743951.1	CAD89161.1	4e-30	32.50%	86%
peptidoglycan recognition protein	bacterial recognition	PGRP-SC2	XP_003743951.1	CAD89187.1	5e-32	31.40%	93%
peptidoglycan recognition protein	pgn degradation and antibacterial activity	PGRP-SB1	XP_003743951.1	CAD89136.1	2e-29	30.60%	90%
peptidoglycan recognition protein	blocking of imd pathway	PGRP-LF	XP_003743951.1	NP_648299.3	5e-37	37.37%	83%
peptidoglycan recognition protein	*activation of imd pathway*	*PGRP-LA*	*Not found*	*AAF50304.2*	*Not found*	*Not found*	*Not found*
Gram-negative binding protein 1	*bacterial and fungal pattern recognition*	*GNBP1*	*Not found*	*Q9NHB0.2*	*Not found*	*Not found*	*Not found*
Gram-negative binding protein 2	*bacterial and fungal pattern recognition*	*GNBP2*	*Not found*	*ACU30172.1*	*Not found*	*Not found*	*Not found*
Gram-negative binding protein 3	*bacterial and fungal pattern recognition*	*GNBP3*	*Not found*	*CAJ18910.1*	*Not found*	*Not found*	*Not found*
c-type lectin 1	*bacterial recognition,*	*DL1*	*Not found*	*AAF53793.1*	*Not found*	*Not found*	*Not found*

	induction of PPO cascade						
c-type lectin 2	*bacterial recognition, induction of PPO cascade*	*DL2*	*Not found*	*NP_001014489.1*	*Not found*	*Not found*	*Not found*
c-type lectin 3 or solute carrier	*bacterial recognition, induction of PPO cascade*	*DL3*	*Not found*	*NP_001014490.1*	*Not found*	*Not found*	*Not found*
galectin 4	several roles have been hypothesized	galectin	XP_003747837.2	ADZ99399.1	2e-22	28.28%	84%
CD109 antigen-like	mark pathogens for phagocytosis	Tep1	XP_028966398.1	CAB87807.1	0.0	30.77%	99%
CD109 antigen-like	mark pathogens	Tep2	XP_028966398.1	CAB87808.1	0.0	32.67%	99%
CD109 antigen-like	mark pathogens	Tep3	XP_028966398.1	AAL39195.1	0.0	32.30%	96%
CD109 antigen-like	mark pathogens	Tep4	XP_028966398.1	NP_523603.2	0.0	30.72%	99%
scavenger receptor class B member 1-like	bacterial and fungal recognition	pes	XP_003742848.1	AHN54246.1	2e-60	27.90%	78%
lysosome membrane protein 2-like	bacterial and fungal recognition	crq	XP_018495037.2	AAF51494.1	7e-70	28.76%	91%
protein draper-like	bacterial and fungal recognition	drpr	XP_028968818.1	NP_477450.1	5e-72	34.12%	86%
scavenger receptor class c, type i	bind to lipoproteins and bacteria	sr-CI	XP_018495712.2	AAW79470.1	3e-07	37.08%	16%
scavenger receptor class c, type ii	bind to lipoproteins and bacteria	sr-CII	XP_003742800.2	AAF58551.1	2e-04	34.83%	14%
scavenger receptor class c, type iii	bind to lipoproteins and bacteria	sr-CIII	XP_028967225.1	AAF37564.1	0.001	26.60%	26%
scavenger receptor class c, type iv	bind to lipoproteins and	sr-CIV	XP_028968216.1	AAF51092.1	9e-05	28.81%	25%

	bacteria						
eater	receptor in phagocytosis and microbial binding	eater	XP_028968540.1	AAF56664.5	1e-23	37.95%	83%
protein draper-like/Nimrod	receptor in phagocytosis and microbial binding	Drp/Nim	XP_028968818.1	AAF53364.2	2e-14	28.30%	61%
**Immune genes involved in SIGNALING**							
spätzle 1B	Toll pathway	spz1-1	XP_028966762.1	NP_733188.1	3e-06	25.32%	62%
spätzle 1Bii	Toll pathway	spz1-2	XP_028966762.1	NP_001138116.1	4e-06	25.32%	52%
spätzle 2, neurotrophin 1	Toll pathway	Spz2	XP_028967867.1	NP_001261417.1	1e-16	27.36%	20%
spätzle 3	Toll pathway	Spz3	XP_003743230.1	NP_609160.2	4e-57	44.83%	37%
spätzle 4	Toll pathway	Spz4	XP_003743230.1	NP_609504.2	3e-26	43.75%	16%
spätzle 5	Toll pathway	Spz5	XP_018497372.1	NP_647753.1	1e-16	39.37%	29%
spätzle 6	Toll pathway	Spz6	XP_018496958.1	NP_611961.1	4e-47	41.71%	83%
protein Toll	Toll pathway	Toll-1	XP_003745335.2	NP_524518.1	2e-91	29.13%	79%
protein Toll	Toll pathway	Toll-1	XP_003741824.2	NP_524518.1	2e-72	25.58%	90%
18 wheeler, Toll-2	Toll pathway	18w	XP_003742102.2	NP_476814.1	0.0	39.26%	83%
Toll-6	Toll pathway	Toll-6	XP_003742102.2	NP_001246766.1	0.0	41.05%	77%
Toll-6	Toll pathway	Toll-6	XP_003738314.1	NP_001246766.1	0.0	40.73%	77%
Toll-7	Toll pathway	Toll-7	XP_003738314.1	NP_523797.1	0.0	41.92%	80%
Tollo, Toll-8	Toll pathway	Tollo	XP_003742102.2	NP_524757.1	0.0	40.91%	90%
tube, interleukin-1 receptor-associated kinase 4	*Toll pathway*	*tub*	*Not found*	*NP_001189164.1*	*Not found*	*Not found*	*Not found*
myeloid differentiation primary response gene	*Toll pathway*	*Myd88pll*	*Not found*	*AAF58953.1*	*5e-19*	*25.56%*	*Not found*
pelle	Toll pathway	pll	XP_018496941.1	AAF56686.1	3e-80	37.14%	91%

cactus	Toll pathway	cact	XP_028967211 .1	AAN10936.1	7e-19	28.17%	55%
cactin	Toll pathway	cactin	XP_028966691 .1	NP_523422.4	0.0	49.92%	82%
pellino	Toll pathway	Pli	XP_003738502. 1	NP_524466.1	0.0	60.70%	93%
TNF-receptor-associated factor 1	Toll pathway	Traf1, Traf4	XP_028967113 .1	AAD34346.1	4e-118	52.00%	75%
TNF-receptor-associated factor 2	Toll pathway	Traf2, Traf6	XP_028967113 .1	AAF46338.1	3e-09	24.18%	30%
TNF-receptor-associated factor 3	Toll pathway	Traf3, Traf-like	XP_028967113 .1	NP_727976.1	3e-20	30.39%	38%
dorsal	Toll pathway	dl	XP_003741763 .1	AAF53611.1	5e-80	48.89%	38%
domeless 1, interleukine JAK/STAT receptor	Jak/stat pathway	dome	XP_003744080 .1	CAD12503.1	6e-24	22.51%	41%
Domeless2	Jak/stat pathway	dome2	XP_003744080 .1	XP_029341036 .1	1e-24	23.89%	33%
hopscotch, Janus kinas	Jak/stat pathway	hops, jak	XP_018496425 .2	NP_511119.2	6e-50	28.67%	47%
signal-transducer and activator of transcription, marelle	Jak/stat pathway	Stat92E	XP_028967482 .1	AAX33462.1	4e-127	45.05%	57%
unpaired 1	*Jak/stat pathway*	*upd1*	*Not found*	*NP_525095.2*	*Not found*	*Not found*	*Not found*
unpaired 2	*Jak/stat pathway*	*Upd2*	*Not found*	*NP_00135688 2.1*	*Not found*	*Not found*	*Not found*
unpaired 3	*Jak/stat pathway*	*Upd3*	*Not found*	*NP_00109701 4.1*	*Not found*	*Not found*	*Not found*
immune deficiency	*Imd pathway*	*imd*	*Not found*	*NP_573394.1*	*Not found*	*Not found*	*Not found*
dFadd	*Imd pathway*	*dFadd*	*Not found*	*NP_651006.1*	*Not found*	*Not found*	*Not found*
death related ced-3, caspase-1	Imd pathway	Dredd	XP_003737022 .1	NP_477249.3	3e-17	28.73%	49%
Relish	Imd pathway	Rel	XP_003741763 .1	NP_477094.1	1e-22	31.70%	26%
TAK1-associated binding protein 2	Imd pathway	Tab2	XP_028966435 .1	NP_611408.2	5e-04	58.33%	2%
TGF-β	Imd pathway	Tak1	XP_028968197	AAF50895.1	2e-78	40.17%	51%

activated Kinase 1			.1				
kenny	*Imd pathway*	*key*	*Not found*	*NP_523856.2*	*Not found*	*Not found*	*Not found*
death-associated inhibitor of apoptosis 2	Imd pathway	Diap2	XP_003741968.1	NP_477127.1	1e-72	31.01%	98%
immune response deficiency 5, IK-β, IKKB, I-kappaB kinase beta	Imd pathway	ird5	XP_028968776.1	NP_524751.3	4e-32	31.37%	44%
hemipterous	Jnk pathway	hep	XP_003745005.1	NP_727661.1	1e-108	57.30%	23%
basket	Jnk pathway	bsk	XP_003743075.1	P92208.1	0.0	85.28%	96%
Jun-related antigen	Jnk pathway	Jra	XP_003739128.1	AAF58845.1	3e-30	31.23%	78%
kayak	Jnk pathway	kay	XP_018496241.1	NP_001027579.1	5e-14	38.52%	20%
Eiger	*Jnk pathway*	*egr*	*Not found*	*AAF58848.2*	*Not found*	*Not found*	*Not found*
**Immune genes involved in RESPONSE**							
Attacin	*antimicrobial peptide*	*att*	*Not found*	*NP_523745.1*	*Not found*	*Not found*	*Not found*
Cecropin	*antimicrobial peptide*	*Cec*	*Not found*	*C0HKQ7.1*	*Not found*	*Not found*	*Not found*
Defensin	*antimicrobial peptide*	*Def*	*Not found*	*ANY27112.1*	*Not found*	*Not found*	*Not found*
Dosocin	*antimicrobial peptide*	*Dro*	*Not found*	*XP_016946682.1*	*Not found*	*Not found*	*Not found*
Metchnikowin	*antimicrobial peptide*	*Mtk*	*Not found*	*AAO72489.1*	*Not found*	*Not found*	*Not found*
Andropin	*antimicrobial peptide*		*Not found*	*P21663.1*	*Not found*	*Not found*	*Not found*
Diptericin	*antimicrobial peptide*		*Not found*	*QER92349.1*	*Not found*	*Not found*	*Not found*
drosomycin	*antimicrobial peptide*	*Drs*	*Not found*	*ANY27466.1*	*Not found*	*Not found*	*Not found*
holotricin	*antimicrobial peptide*		*Not found*	*XP_051861657.1*	*Not found*	*Not found*	*Not found*
bomanin	*antimicrobial peptide*		*Not found*	*A1ZB62.1*	*Not found*	*Not found*	*Not found*
thaumatin-like protein	*antimicrobial*		*Not found*	*XP_001942718.2*	*Not found*	*Not found*	*Not found*
thaumatin-like protein 1b	*antimicrobial*		*Not found*	*XP_001942572.1*	*Not found*	*Not found*	*Not found*
thaumatin-like protein 1	*antimicrobial*		*Not found*	*XP_003248856.4*	*Not found*	*Not found*	*Not found*

uncharacterized LOC100162111, thaumatin family	*antimicrobial*		*Not found*	*NP_001155516*	*Not found*	*Not found*	*Not found*
TLP-PA-domain protein	*antimicrobial*		*Not found*	*NP_001156304.1*	*Not found*	*Not found*	*Not found*
Pathogenesis-related protein 5-like	*antimicrobial*		*Not found*	*NP_001313585.1*	*Not found*	*Not found*	*Not found*
lysozyme X, i-type	microbial degradation	LysX	XP_003743605.1	CAL85493.1	2e-30	40.15%	95%
lysozyme B, i-type	microbial degradation	LysB	XP_003743605.1	NP_001261245.1	3e-30	41.94%	87%
lysozyme, i-type	microbial degradation	LysP	XP_018495125.1	NP_476828.1	4e-31	45.31%	97%
Lysozyme E	microbial degradation	LysE	XP_003743605.1	CAA80228	4e-30	38.57%	97%
Lysozyme D	microbial degradation	LysD	XP_003743605.1	NP_476823.1	1e-30	39.29%	97%
Lysozyme E	microbial degradation	LysE	XP_003743605.1	NP_476827.2	5e-29	41.13%	87%
Lysozyme S	microbial degradation	LysS	XP_003743605.1	NP_476829.1	4e-34	46.34%	86%
Lysozyme E	microbial degradation	lysozyme, i-type	XP_003742436.1	ACD99447.1	1e-25	41.10%	78%
Lysozyme	microbial degradation	lysozyme, i-type	XP_003742436.1	NP_611164.3	1e-17	34.35%	79%
Lysozyme	microbial degradation	lysozyme, i-type	XP_003742436.1	NP_611163.2	5e-23	36.13%	91%
chitinase-like protein 4, flocculation protein	fungal degradation	Cht2	XP_003739697.1	NP_001261282.1	2e-111	47.40%	73%
chitinase-like protein 2, mucin	fungal degradation	Cht4	XP_003739697.1	NP_524962.2	5e-107	43.99%	90%
chitinase-like protein 5, endochitinase	fungal degradation	Cht5	XP_018495001.1	NP_650314.1	2e-160	41.09%	96%
chitinase-like protein 6, flocculation protein	fungal degradation	Cht6	XP_028968641.1	NP_001245602.1	1e-176	50.84%	16%
chitinase-like protein 7, chitinase 10	fungal degradation	Cht7	XP_028966665.1	NP_647768.3	0.0	53.13%	99%
chitinase 3-	fungal	Cht7	XP_003739697	NP_647768.3	2e-90	41.90%	78%

like	degradation		.1				
Chitinase 6, flocculation protein FLO11	fungal degradation	Cht6	XP_028968641.1	NP_001245599.1	4e-180	50.84%	43%
idgf	fungal degradation	idgf6	XP_018495001.1	NP_001286499.1	3e-40	29.11%	93%
prophenoloxidase 1	*prophenoloxidase response*	*PPO1*	*Not found*	*NP_476812.1*	*Not found*	*Not found*	*Not found*
prophenoloxidase 2	*prophenoloxidase response*	*PPO2*	*Not found*	*NP_610443.1*	*Not found*	*Not found*	*Not found*
Phenoloxidase-activating factor 2 (tryptase like)	phenoloxidase activation	PAF2, PPAF2	XP_003744063.1	AAO24923.1	7e-77	38.97%	77%
Phenoloxidase-activating factor 2	phenoloxidase activation	PAF2, PPAF2	XP_018497577.1	AAO24923.1	1e-72	44.70%	64%
serine protease-like precursor	phenoloxidase activation	SP	XP_018494399.1	NP_001097766.1	2e-45	38.76%	55%
hemocytin	cell aggregation	Hmct, hemolectin	XP_028967464.1	NP_001261809.1	6e-157	32.22%	71%
nitric oxide synthase	production of nitric oxide, a toxic gas	Nos	XP_018494079.1	NP_001027243.2	5e-73	28.49	59%
transglutaminase	clotting	Tg	XP_028966952.1	NP_609174.1	3e-127	33.58%	90%
