# Supplementary material for: Immune-Related Genes in the Honey Bee Mite Varroa destructor (Acarina, Parasitidae)
Source: Insects. 2025 Mar 28;16(4):356. doi: 10.3390/insects16040356 (PMC12027997; doi:10.3390/insects16040356)
Supplement: Supplementary file 1 [file insects-16-00356-s001.zip › Table S3.pdf]

**Table S3. Immune genes involved in recognition, signaling and response in *Tetranychus urticae***

| Gene Name | Role | Gene Symbol | Tetranychus urticae | Drosophila melanogaster | E-Value | identity | Coverage |
|---|---|---|---|---|---|---|---|
| **Immune genes involved in RECOGNITION** | | | | | | | |
| peptidoglycan recognition protein | bacterial recognition | PGRP-LC | XP_015788186.1 | AAF50302.3 | 5e-14 | 28.99 % | 27% |
| *peptidoglycan recognition protein* | *activation of PPO cascade and autophagy* | *PGRP-LE* | *Not found* | *NP_573078.1* | *Not found* | *Not found* | *Not found* |
| peptidoglycan recognition protein | bacterial recognition | PGRP-SA | XP_015788186.1 | AAF48056.1 | 7e-15 | 29.32 % | 65% |
| *peptidoglycan recognition protein* | *bacterial recognition* | *PGRP-SD* | *Not found* | *CAD89193.1* | *Not found* | *Not found* | *Not found* |
| *peptidoglycan recognition protein* | *bacterial recognition* | *PGRP-LB* | *Not found* | *NP_650079.1* | *Not found* | *Not found* | *Not found* |
| *peptidoglycan recognition protein* | *bacterial recognition* | *PGRP-SC1a* | *Not found* | *CAD89161.1* | *Not found* | *Not found* | *Not found* |
| *peptidoglycan recognition protein* | *bacterial recognition* | *PGRP-SC2* | *Not found* | *CAD89187.1* | *Not found* | *Not found* | *Not found* |
| peptidoglycan recognition protein | pgn degradation and antibacterial activity | PGRP-SB1 | XP_015788186.1 | CAD89136.1 | 9e-18 | 31.65 % | 82% |
| peptidoglycan recognition protein | blocking of imd pathway | PGRP-LF | XP_015788186.1 | NP_648299.3 | 6e-20 | 29.11 % | 42% |
| *peptidoglycan recognition protein* | *activation of imd pathway* | *PGRP-LA* | *Not found* | *AAF50304.2* | *Not found* | *Not found* | *Not found* |
| *Gram-negative binding protein 1* | *bacterial and fungal pattern recognition* | *GNBP1* | *Not found* | *Q9NHB0.2* | *Not found* | *Not found* | *Not found* |
| *Gram-negative binding protein 2* | *bacterial and fungal pattern recognition* | *GNBP2* | *Not found* | *ACU30172.1* | *Not found* | *Not found* | *Not found* |
| *Gram-negative binding protein 3* | *bacterial and fungal pattern* | *GNBP3* | *Not found* | *CAJ18910.1* | *Not found* | *Not found* | *Not found* |

| | | | | | | | |
|---|---|---|---|---|---|---|---|
| | *recognition* | | | | | | |
| c-type lectin 1 | bacterial recognition, induction of PPO cascade | DL1 | XP_015784111.1 | AAF53793.1 | 2e-04 | 29.55 % | 60% |
| *c-type lectin 2* | *bacterial recognition, induction of PPO cascade* | *DL2* | *Not found* | *NP_001014489.1* | *Not found* | *Not found* | *Not found* |
| *c-type lectin 3 or solute carrier* | *bacterial recognition, induction of PPO cascade* | *DL3* | *Not found* | *NP_001014490.1* | *Not found* | *Not found* | *Not found* |
| galectin 4 | several roles have been hypothesized | galectin | XP_015791541.1 | ADZ99399.1 | 1e-34 | 29.82 % | 83% |
| CD109 antigen-like | mark pathogens for phagocytosis | Tep1 | XP_015784902.1 | CAB87807.1 | 0.0 | 32.00 % | 99% |
| CD109 antigen-like | mark pathogens | Tep2 | XP_015784901.1 | CAB87808.1 | 0.0 | 35.83 % | 99% |
| CD109 antigen-like | mark pathogens | Tep3 | XP_015784902.1 | AAL39195.1 | 0.0 | 30.67 % | 95% |
| CD109 antigen-like | mark pathogens | Tep4 | XP_015784902.1 | NP_523603.2 | 0.0 | 31.01 % | 99% |
| scavenger receptor class B member 1-like | bacterial and fungal recognition | pes | XP_015782139.1 | AHN54246.1 | 8e-62 | 30.39 % | 71% |
| lysosome membrane protein 2-like | bacterial and fungal recognition | crq | XP_015791952.1 | AAF51494.1 | 8e-69 | 27.99 % | 93% |
| protein draper-like | bacterial and fungal recognition | drpr | XP_015784755.1 | NP_477450.1 | 1e-56 | 30.51 % | 86% |
| scavenger receptor class c, type i | bind to lipoproteins and bacteria | sr-CI | XP_015787528.1 | AAW79470.1 | 7e-32 | 25.71 % | 72% |
| scavenger receptor class c, type ii | bind to lipoproteins and bacteria | sr-CII | XP_015787528.1 | AAF58551.1 | 7e-24 | 25.59 % | 60% |

| | | | | | | | |
|---|---|---|---|---|---|---|---|
| scavenger receptor class c, type iii | bind to lipoproteins and bacteria | sr-CIII | XP_015787528.1 | AAF37564.1 | 3e-09 | 21.34 % | 75% |
| scavenger receptor class c, type iv | bind to lipoproteins and bacteria | sr-CIV | XP_015787528.1 | AAF51092.1 | 5e-21 | 24.03 % | 86% |
| eater | receptor in phagocytosis and microbial binding | eater | XP_015789655.1 | AAF56664.5 | 9e-18 | 35.20 % | 72% |
| protein draper-like | receptor in phagocytosis and microbial binding | drp | XP_015789655.1 | AAF53364.2 | 1e-09 | 32.89 % | 63% |
| **Immune genes involved in SIGNALING** | | | | | | | |
| spätzle 1B | Toll pathway | spz1-1 | XP_015786692.1 | NP_733188.1 | 6e-13 | 29.41 % | 62% |
| spätzle 1Bii | Toll pathway | spz1-2 | XP_015786692.1 | NP_001138116.1 | 7e-13 | 30.67 % | 52% |
| spätzle 2, neurotrophin 1 | Toll pathway | Spz2 | XP_015786820.1 | NP_001261417.1 | 1e-23 | 41.41 % | 9% |
| spätzle 3 | Toll pathway | Spz3 | XP_015788003.1 | NP_609160.2 | 7e-54 | 72.38 % | 24% |
| spätzle 4 | Toll pathway | Spz4 | XP_015786555.1 | NP_609504.2 | 5e-62 | 75.00 % | 29% |
| spätzle 5 | Toll pathway | Spz5 | XP_015790281.1 | NP_647753.1 | 2e-21 | 42.11 % | 27% |
| spätzle 6 | Toll pathway | Spz6 | XP_015786866.1 | NP_611961.1 | 7e-31 | 68.92 % | 50% |
| protein Toll | Toll pathway | Toll-1 | XP_015785737.1 | NP_524518.1 | 7e-46 | 23.00 % | 77% |
| protein Toll | Toll pathway | Toll-1 | XP_015790447.1 | NP_524518.1 | 2e-29 | 45.31 % | 57% |
| 18 wheeler, Toll-2 | Toll pathway | 18w | XP_015785737.1 | NP_476814.1 | 0.0 | 37.21 % | 81% |
| Toll-6 | Toll pathway | Toll-6 | XP_015785737.1 | NP_001246766.1 | 0.0 | 37.80 % | 77% |
| Toll-6 | Toll pathway | Toll-6 | XP_015793245.1 | NP_001246766.1 | 0.0 | 42.92 % | 57% |
| Toll-7 | Toll pathway | Toll-7 | XP_015785737.1 | NP_523797.1 | 0.0 | 38.52 % | 78% |
| Tollo, Toll-8 | Toll pathway | Tollo | XP_015785737.1 | NP_524757.1 | 0.0 | 37.54 % | 89% |
| tube, interleukin-1 receptor-associated kinase 4 | Toll pathway | tub | XP_015795654.1 | NP_001189164.1 | 3e-10 | 29.60 % | 27% |

| | | | | | | | |
|---|---|---|---|---|---|---|---|
| myeloid differentiation primary response gene | Toll pathway | Myd88pll | XP_015786667.1 | AAF58953.1 | 4e-28 | 27.78 % | 61% |
| pelle | Toll pathway | pll | XP_015785544.1 | AAF56686.1 | 2e-56 | 38.53 % | 69% |
| cactus | Toll pathway | cact | XP_015788233.1 | AAN10936.1 | 9e-40 | 38.57 % | 44% |
| cactin | Toll pathway | cactin | XP_015790419.1 | NP_523422.4 | 3e-167 | 47.07 % | 81% |
| pellino | Toll pathway | Pli | XP_015784371.1 | NP_524466.1 | 0.0 | 66.59 % | 93% |
| TNF-receptor-associated factor 1 | Toll pathway | Traf1, Traf4 | XP_015792200.1 | AAD34346.1 | 3e-10 | 28.85 % | 21% |
| TNF-receptor-associated factor 2 | Toll pathway | Traf2, Traf6 | XP_015782873.1 | AAF46338.1 | 4e-09 | 27.03 % | 23% |
| TNF-receptor-associated factor 3 | Toll pathway | Traf3, Traf-like | XP_015787576.1 | NP_727976.1 | 0.002 | 25.93 | 26% |
| dorsal | Toll pathway | dl | XP_015786990.1 | AAF53611.1 | 3e-143 | 66.78 % | 43% |
| domeless 1, interleukine JAK/STAT receptor | Jak/stat pathway | dome | XP_025016179.1 | CAD12503.1 | 1e-14 | 21.74 % | 25% |
| Domeless2 | Jak/stat pathway | dome2 | XP_015782404.1 | XP_029341036.1 | 2e-15 | 20.99 % | 48% |
| hopscotch, Janus kinas | Jak/stat pathway | hops, jak | XP_015794466.1 | NP_511119.2 | 2e-59 | 24.35 % | 94% |
| signal-transducer and activator of transcription, marelle | Jak/stat pathway | Stat92E | XP_015793454.1 | AAX33462.1 | 5e-104 | 40.28 % | 62% |
| *unpaired 1* | *Jak/stat pathway* | *upd1* | *Not found* | *NP_525095.2* | *Not found* | *Not found* | *Not found* |
| *unpaired 2* | *Jak/stat pathway* | *Upd2* | *Not found* | *NP_001356882.1* | *Not found* | *Not found* | *Not found* |
| *unpaired 3* | *Jak/stat pathway* | *Upd3* | *Not found* | *NP_001097014.1* | *Not found* | *Not found* | *Not found* |
| *immune deficiency* | *Imd pathway* | *imd* | *Not found* | *NP_573394.1* | *Not found* | *Not found* | *Not found* |
| *dFadd* | *Imd pathway* | *dFadd* | *Not found* | *NP_651006.1* | *Not found* | *Not found* | *Not found* |
| death related ced-3, caspase-1 | Imd pathway | Dredd | XP_015781719.1 | NP_477249.3 | 4e-16 | 26.51 % | 56% |
| Relish | Imd pathway | Rel | XP_015784176.1 | NP_477094.1 | 5e-68 | 43.43 % | 32% |

| | | | | | | | |
|---|---|---|---|---|---|---|---|
| TAK1-associated binding protein 2 | Imd pathway | Tab2 | XP_015790115.1 | NP_611408.2 | 7e-04 | 48.15 % | 3% |
| TGF-β activated Kinase 1 | Imd pathway | Tak1 | XP_015784617.1 | AAF50895.1 | 7e-76 | 36.68 % | 50% |
| *kenny* | *Imd pathway* | *key* | *Not found* | *NP_523856.2* | *Not found* | *Not found* | *Not found* |
| death-associated inhibitor of apoptosis 2 | Imd pathway | Diap2 | XP_015783081.1 | NP_477127.1 | 6e-27 | 33.17 % | 65% |
| immune response deficiency 5, IK-β, IKKB, I-kappaB kinase beta | Imd pathway | ird5 | XP_015791606.1 | NP_524751.3 | 1e-27 | 29.31 % | 47% |
| hemipterous | Jnk pathway | hep | XP_015794871.1 | NP_727661.1 | 1e-118 | 56.31 % | 25% |
| basket | Jnk pathway | bsk | XP_015784065.1 | P92208.1 | 0.0 | 82.83 % | 97% |
| Jun-related antigen | Jnk pathway | Jra | XP_015787799.1 | AAF58845.1 | 4e-30 | 64.20 % | 28% |
| kayak | Jnk pathway | kay | XP_015785509.2 | NP_001027579.1 | 3e-09 | 30.36 % | 18% |
| *Eiger* | *Jnk pathway* | *egr* | *Not found* | *AAF58848.2* | *Not found* | *Not found* | *Not found* |
| **Immune genes involved in RESPONSE** | | | | | | | |
| *Attacin* | *antimicrobial peptide* | *att* | *Not found* | *NP_523745.1* | *Not found* | *Not found* | *Not found* |
| *Cecropin* | *antimicrobial peptide* | *Cec* | *Not found* | *C0HKQ7.1* | *Not found* | *Not found* | *Not found* |
| *Defensin* | *antimicrobial peptide* | *Def* | *Not found* | *ANY27112.1* | *Not found* | *Not found* | *Not found* |
| *Dosocin* | *antimicrobial peptide* | *Dro* | *Not found* | *XP_016946682.1* | *Not found* | *Not found* | *Not found* |
| *Metchnikowin* | *antimicrobial peptide* | *Mtk* | *Not found* | *AAO72489.1* | *Not found* | *Not found* | *Not found* |
| *Andropin* | *antimicrobial peptide* | | *Not found* | *P21663.1* | *Not found* | *Not found* | *Not found* |
| *Diptericin* | *antimicrobial peptide* | | *Not found* | *QER92349.1* | *Not found* | *Not found* | *Not found* |

| | | | | | | | |
|---|---|---|---|---|---|---|---|
| *drosomycin* | *antimicrobial peptide* | *Drs* | *Not found* | *ANY27466.1* | *Not found* | *Not found* | *Not found* |
| *holotricin* | *antimicrobial peptide* | | *Not found* | *XP_051861657.1* | *Not found* | *Not found* | *Not found* |
| *bomanin* | *antimicrobial peptide* | | *Not found* | *A1ZB62.1* | *Not found* | *Not found* | *Not found* |
| *thaumatin-like protein* | *antimicrobial* | | *Not found* | *XP_001942718.2* | *Not found* | *Not found* | *Not found* |
| *thaumatin-like protein 1b* | *antimicrobial* | | *Not found* | *XP_001942572.1* | *Not found* | *Not found* | *Not found* |
| *thaumatin-like protein 1* | *antimicrobial* | | *Not found* | *XP_003248856.4* | *Not found* | *Not found* | *Not found* |
| *uncharacterized LOC100162111, thaumatin family* | *antimicrobial* | | *Not found* | *NP_001155516* | *Not found* | *Not found* | *Not found* |
| *TLP-PA-domain protein* | *antimicrobial* | | *Not found* | *NP_001156304.1* | *Not found* | *Not found* | *Not found* |
| *Pathogenesis-related protein 5-like* | *antimicrobial* | | *Not found* | *NP_001313585.1* | *Not found* | *Not found* | *Not found* |
| lysozyme X, i-type | microbial degradation | LysX | XP_015785788.1 | CAL85493.1 | 6e-27 | 38.17 % | 90% |
| lysozyme B, i-type | microbial degradation | LysB | XP_015785788.1 | NP_001261245.1 | 2e-33 | 42.31 % | 91% |
| lysozyme, i-type | microbial degradation | LysP | XP_015785788.1 | NP_476828.1 | 4e-31 | 40.46 % | 91% |
| Lysozyme E | microbial degradation | LysE | XP_015785788.1 | CAA80228 | 1e-31 | 40.77 % | 91% |
| Lysozyme D | microbial degradation | LysD | XP_015785788.1 | NP_476823.1 | 2e-32 | 41.54 % | 91% |
| Lysozyme E | microbial degradation | LysE | XP_015785788.1 | NP_476827.2 | 3e-33 | 41.54 % | 91% |
| Lysozyme S | microbial degradation | LysS | XP_015785788.1 | NP_476829.1 | 5e-26 | 36.15 % | 91% |
| Lysozyme E | microbial degradation | lysozyme, i-type | XP_015795982.1 | ACD99447.1 | 3e-25 | 41.60 % | 73% |
| Lysozyme | microbial degradation | lysozyme, i-type | XP_015795983.1 | NP_611164.3 | 3e-22 | 40.80 % | 76% |
| Lysozime | microbial degradation | lysozyme, i-type | XP_015795982.1 | NP_611163.2 | 4e-22 | 39.42 % | 68% |
| chitinase-like protein 4, flocculation protein | fungal degradation | Cht2 | XP_015789865.1 | NP_001261282.1 | 3e-117 | 51.65 % | 73% |
| chitinase-like protein 2, | fungal degradation | Cht4 | XP_015789865.1 | NP_524962.2 | 3e-115 | 40.35 % | 95% |

| | | | | | | | |
|---|---|---|---|---|---|---|---|
| mucin | | | | | | | |
| chitinase-like protein 5, endochitinase | fungal degradation | Cht5 | NP_001310073.1 | NP_650314.1 | 4e-168 | 46.84 % | 93% |
| chitinase-like protein 6, flocculation protein | fungal degradation | Cht6 | XP_015788876.1 | NP_001245602.1 | 3e-153 | 53.67 % | 10% |
| chitinase-like protein 7, chitinase 10 | fungal degradation | Cht7 | XP_015795759.1 | NP_647768.3 | 0.0 | 57.42 % | 89% |
| chitinase 3-like | fungal degradation | Cht7 | XP_015795758.1 | NP_647768.3 | 0.0 | 57.42 % | 89% |
| Chitinase 6, flocculation protein FLO11 | fungal degradation | Cht6 | XP_015788876.1 | NP_001245599.1 | 0.0 | 51.07 % | 40% |
| idgf | fungal degradation | idgf6 | XP_015788876.1 | NP_001286499.1 | 2e-43 | 27.87 % | 99% |
| *prophenoloxidase 1* | *prophenoloxidase response* | *PPO1* | *Not found* | *NP_476812.1* | *Not found* | *Not found* | *Not found* |
| *prophenoloxidase 2* | *prophenoloxidase response* | *PPO2* | *Not found* | *NP_610443.1* | *Not found* | *Not found* | *Not found* |
| Phenoloxidase-activating factor 2 (tryptase like) | phenoloxidase activation | PAF2, PPAF2 | XP_025017228.1 | AAO24923.1 | 3e-82 | 39.30 % | 86% |
| Phenoloxidase-activating factor 2 | phenoloxidase activation | PAF2, PPAF2 | XP_015790479.1 | AAO24923.1 | 3e-77 | 37.75 % | 81% |
| serine protease-like precursor | phenoloxidase activation | SP | XP_015784864.1 | NP_001097766.1 | 1e-53 | 33.16 % | 83% |
| hemocytin | cell aggregation | Hmct, hemolectin | XP_015790438.1 | NP_001261809.1 | 1e-06 | 31.43 % | 4% |
| nitric oxide synthase | production of nitric oxide, a toxic gas | Nos | XP_015793516.1 | NP_001027243.2 | 0.0 | 58.84 % | 96% |
| transglutaminase | clotting | Tg | XP_015793580.1 | NP_609174.1 | 0.0 | 41.84 % | 89% |
