# Supplementary material for: Immune-Related Genes in the Honey Bee Mite Varroa destructor (Acarina, Parasitidae)
Source: Insects. 2025 Mar 28;16(4):356. doi: 10.3390/insects16040356 (PMC12027997; doi:10.3390/insects16040356)
Supplement: Supplementary file 1 [file insects-16-00356-s001.zip › Table S4.pdf]

Table S4. Results of BLASTp searches against Genbank nr protein database using lectin DL3 putative homolog from Varroa as query

Target Species	Query	Best Hit	E-Value	identity	Coverage
I. scapularis	XP_022646715.1	XP_029831539.2	2e-39	33.78%	88%
G. occidentalis	XP_022646715.1	XP_018494856.1	3e-98	42.25%	93%
T. urticae	XP_022646715.1	XP_015787611.1	2e-06	26.89%	32%[1]

[1] This low coverage suggest no homology with lectin DL3. Indeed, protein description for this match was sushi, von Willebrand factor type A, EGF and pentraxin domain-containing protein 1
