# Supplementary material for: Immune-Related Genes in the Honey Bee Mite Varroa destructor (Acarina, Parasitidae)
Source: Insects. 2025 Mar 28;16(4):356. doi: 10.3390/insects16040356 (PMC12027997; doi:10.3390/insects16040356)
Supplement: Supplementary file 1 [file insects-16-00356-s001.zip › Table S5.pdf]

Table S5. Results of BLASTp searches against Genbank nr protein database using tube and Myd88 putative homologs from Tetranychus urticae as query

Target Species	Gene Name	Gene Symbol	Query	Best Hit	E-Value	identity	Coverage
V. destructor	tube, interleukin-1 receptor-associated kinase 4	tub	XP_015795654.1	XP_022667915.1	3e-17	28.16%	60%
	myeloid differentiation primary response gene	Myd88	XP_015786667.1	XP_022664419.1	1e-42	38.40%	66%
G. occidentalis	tube, interleukin-1 receptor-associated kinase 4	tub	XP_015795654.1	XP_003745554.2	5e-39	38.16%	44%
	myeloid differentiation primary response gene	Myd88	XP_015786667.1	XP_003747371.1	1e-22	26.76%	62%
I. scapularis	tube, interleukin-1 receptor-associated kinase 4	tub	XP_015795654.1	XP_002404763.4	2e-73	31.36%	98%
	myeloid differentiation primary response gene	Myd88	XP_015786667.1	EEC12280.1	2e-46	35.94%	62%
