# Supplementary material for: Immune-Related Genes in the Honey Bee Mite Varroa destructor (Acarina, Parasitidae)
Source: Insects. 2025 Mar 28;16(4):356. doi: 10.3390/insects16040356 (PMC12027997; doi:10.3390/insects16040356)
Supplement: Supplementary file 1 [file insects-16-00356-s001.zip › Table S6.pdf]

Table S6. Results of BLASTp searches against Genbank nr protein database using p47 putative homologs from Ixodes scapularis as query

Target Species	Query	Best Hit	E-Value	Identity (%)	Coverage (%)
V. destructor	XP_040073288.2	XP_022672598.1	4e-107	44.63	97
G. occidentalis	XP_040073288.2	XP_003746458.1	3e-99	45.77	97
T.urticae	XP_040073288.2	XP_015781608.1	3e-89	46.65	79
