# Supplementary material for: Immune-Related Genes in the Honey Bee Mite Varroa destructor (Acarina, Parasitidae)
Source: Insects. 2025 Mar 28;16(4):356. doi: 10.3390/insects16040356 (PMC12027997; doi:10.3390/insects16040356)
Supplement: Supplementary file 1 [file insects-16-00356-s001.zip › Table S7.pdf]

Table S7. Ctenidin-like proteins identified in *V. destructor, Galendromus occidentalis* and *Tetranychus urticae*

Genbank protein accession	Description	Organism	Best hit accession	E-Value	Identity	Coverage	Length (AA)
**Ctenidin-like proteins identified in V. destructor**							
XP_065305715.1	ctenidin-1-like	*Dermacentor albipictus*	Not found	Not found	Not found	Not found	Not found
XP_064458063.1	ctenidin-3-like	*Ornithodoros turicata*	Not found	Not found	Not found	Not found	Not found
XP_064457183.1	ctenidin-3-like	*Ornithodoros turicata*	Not found	Not found	Not found	Not found	Not found
XP_050051926.1	ctenidin-3-like	*Dermacentor andersoni*	XP_022650280.1	*0.002*	*47.73%*	33%	285
XP_054153978.1	ctenidin-1-like	*Oppia nitens*	Not found	Not found	Not found	Not found	Not found
XP_049514476.1	ctenidin-3-like	*Dermacentor silvarum*	XP_022650280.1	*6e-04*	*48.78%*	38%	285
XP_037576217.1	ctenidin-1-like	*Dermacentor silvarum*	XP_022672922.1	*9e-12*	*53.97%*	59%	115
XP_037521698.1	ctenidin-3-like	*Rhipicephalus sanguineus*	XP_022672922.1	*2e-10*	*53.06%*	36%	115
XP_037517623.1	ctenidin-3-like	*Rhipicephalus sanguineus*	Not found	Not found	Not found	Not found	Not found
XP_037525838.1	ctenidin-3-like	*Rhipicephalus sanguineus*	Not found	Not found	Not found	Not found	Not found
XP_037511163.1	ctenidin-1-like	*Rhipicephalus sanguineus*	XP_022672922.1	*8e-08*	*57.14%*	41%	115
XP_037505847.1	ctenidin-1	*Rhipicephalus sanguineus*	Not found	Not found	Not found	Not found	Not found
Ctenidin-like proteins identified in G. occidentalis							
XP_065305715.1	ctenidin-1-like	*Dermacentor albipictus*	Not found	Not found	Not found	Not found	Not found
XP_064458063.1	ctenidin-3-like	*Ornithodoros turicata*	Not found	Not found	Not found	Not found	Not found

XP_064457183.1	ctenidin-3-like	*Ornithodoros turicata*	Not found	Not found	Not found	Not found	Not found
XP_050051926.1	ctenidin-3-like	*Dermacentor andersoni*	XP_003737597.1	*0.002*	*47.93%*	36%	173
XP_054153978.1	ctenidin-1-like	*Oppia nitens*	Not found	Not found	Not found	Not found	Not found
XP_049514476.1	ctenidin-3-like	*Dermacentor silvarum*	XP_003737597.1	*3e-05*	*40%*	46%	173
XP_037576217.1	ctenidin-1-like	*Dermacentor silvarum*	XP_003747423.1	*2e-14*	*50%*	47%	105
XP_037521698.1	ctenidin-3-like	*Rhipicephalus sanguineus*	XP_003747421.1	*5e-15*	*50.75%*	49%	106
XP_037517623.1	ctenidin-3-like	*Rhipicephalus sanguineus*	Not found	Not found	Not found	Not found	Not found
XP_037525838.1	ctenidin-3-like	*Rhipicephalus sanguineus*	Not found	Not found	Not found	Not found	Not found
XP_037511163.1	ctenidin-1-like	*Rhipicephalus sanguineus*	XP_003747422.1	*1e-07*	*48.78%*	40%	105
XP_037505847.1	ctenidin-1	*Rhipicephalus sanguineus*	Not found	Not found	Not found	Not found	Not found
Ctenidin-like proteins identified in T. urticae							
XP_065305715.1	ctenidin-1-like	*Dermacentor albipictus*	Not found	Not found	Not found	Not found	Not found
XP_064458063.1	ctenidin-3-like	*Ornithodoros turicata*	Not found	Not found	Not found	Not found	Not found
XP_064457183.1	ctenidin-3-like	*Ornithodoros turicata*	Not found	Not found	Not found	Not found	Not found
XP_050051926.1	ctenidin-3-like	*Dermacentor andersoni*	Not found	Not found	Not found	Not found	Not found
XP_054153978.1	ctenidin-1-like	*Oppia nitens*	Not found	Not found	Not found	Not found	Not found
XP_049514476.1	ctenidin-3-like	*Dermacentor silvarum*	Not found	Not found	Not found	Not found	Not found

XP_037576217.1	ctenidin-1-like	*Dermacentor silvarum*	Not found	Not found	Not found	Not found	Not found
XP_037521698.1	ctenidin-3-like	*Rhipicephalus sanguineus*	Not found	Not found	Not found	Not found	Not found
XP_037517623.1	ctenidin-3-like	*Rhipicephalus sanguineus*	Not found	Not found	Not found	Not found	Not found
XP_037525838.1	ctenidin-3-like	*Rhipicephalus sanguineus*	Not found	Not found	Not found	Not found	Not found
XP_037511163.1	ctenidin-1-like	*Rhipicephalus sanguineus*	Not found	Not found	Not found	Not found	Not found
XP_037505847.1	ctenidin-1	*Rhipicephalus sanguineus*	Not found	Not found	Not found	Not found	Not found
